# GDE6 promotes progenitor identity in the vertebrate neural tube

**DOI:** 10.3389/fnins.2023.1047767

**Published:** 2023-03-21

**Authors:** Madeline McKean, Francesca R. Napoli, Tahira Hasan, Thea Joseph, Alison Wheeler, Katherine Beebe, Stephanie Soriano-Cruz, Minori Kawano, Clinton Cave

**Affiliations:** Neuroscience Program, Middlebury College, Middlebury, VT, United States

**Keywords:** GDE6, neural tube development, patterning, neurogenesis, hyperplasia

## Abstract

The generation of neurons in the central nervous system is a complex, stepwise process necessitating the coordinated activity of mitotic progenitors known as radial glia. Following neural tube closure, radial glia undergo a period of active proliferation to rapidly expand their population, creating a densely packed neurepithelium. Simultaneously, radial glia positioned across the neural tube are uniquely specified to produce diverse neuronal sub-types. Although these cellular dynamics are well studied, the molecular mechanisms governing them are poorly understood. The six-transmembrane Glycerophosphodiester Phosphodiesterase proteins (GDE2, GDE3, and GDE6) comprise a family of cell-surface enzymes expressed in the embryonic nervous system. GDE proteins can release Glycosylphosphatidylinositol-anchored proteins from the cell surface *via* cleavage of their lipid anchor. GDE2 has established roles in motor neuron differentiation and oligodendrocyte maturation, and GDE3 regulates oligodendrocyte precursor cell proliferation. Here, we describe a role for GDE6 in early neural tube development. Using RNAscope, we show that *Gde6* mRNA is expressed by ventricular zone progenitors in the caudal neural tube. Utilizing in-ovo electroporation, we show that GDE6 overexpression promotes neural tube hyperplasia and ectopic growths of the neurepithelium. At later stages, electroporated embryos exhibit an expansion of the ventral patterning domains accompanied by reduced cross-repression. Ultimately, electroporated embryos fail to produce the full complement of post-mitotic motor neurons. Our findings indicate that GDE6 overexpression significantly affects radial glia function and positions GDE6 as a complementary factor to GDE2 during neurogenesis.

## Introduction

During embryogenesis, the central nervous system arises from a unique pseudostratified neurepithelium called the neural tube (Paridaen and Huttner, 2014). The neural tube is an ectodermal derivative initially comprised of highly mitotic cells known as radial glia. Radial glia are bipolar progenitor cells with elongated processes that connect to the apical and basal surfaces of the neural tube (Malatesta et al., 2000; Hansen et al., 2010). Radial glia will generate most of the post-mitotic cells in the central nervous system (Jessell, 2000). Radial glia must balance their proliferation (how fast they proceed through the cell cycle) and whether they expand their population (symmetric divisions where both daughter cells remain radial glia) or produce neurons (asymmetric divisions producing one radial glia and one neuron) (Franco and Müller, 2013). Unique neuronal subtypes are produced throughout the neural tube; therefore, individual radial glia situated at distinct points along the neurepithelium will commit to generating specific lineages. Radial glia patterning is controlled by organizing centers that release diffusible morphogens across the neural tube. In the caudal neural tube, which will eventually give rise to the spinal cord, proteins from the Bone Morphogenetic Protein (BMP) and Wnt superfamilies are released dorsally from the roof plate; while Sonic Hedgehog (Shh) is released ventrally from the floor plate (Briscoe et al., 2000; Ribes et al., 2010). Additionally, retinoic acid enters the neural tube laterally, released by somites in the paraxial mesoderm (Novitch et al., 2003). Individual radial glia positioned at different points along the neural tube will transduce their respective morphogen concentrations, each adopting exclusive transcriptional profiles. Once specified, these progenitors subsequently produce distinct post-mitotic neuronal subtypes (Alaynick et al., 2011). While the broad cellular dynamics of these developmental stages are well characterized, their molecular regulators are not fully understood.

Over the past decade, family members of the six-transmembrane Glycerophosphodiester Phosphodiesterases (GDEs) have been identified as integral molecules in the context of cell proliferation and differentiation during neurodevelopment (Rao and Sockanathan, 2005; Yan et al., 2009; Sabharwal et al., 2011; Rodriguez et al., 2012; Choi et al., 2020b). These proteins share a six-transmembrane topology with intracellular N- and C-termini and an extracellular enzymatic (GDPD) domain. Expressed on the cell surface, GDE proteins regulate the release of Glycosylphosphatidylinositol-anchored proteins (GPI-APs) (Park et al., 2013). GPI-anchorage is a common post-translational modification that tethers proteins to the cell surface with a lipid anchor, and GPI-APs have been identified as components in numerous signaling pathways (Chatterjee and Mayor, 2001; Maeda and Kinoshita, 2011). GDEs cleave within the GPI-anchor, releasing the protein into the extracellular space. The release of GPI-APs can produce cell autonomous effects (due to the loss of a surface protein) and non-cell autonomous effects (*via* the conditioning of the extracellular matrix with cleaved GPI-APs). These findings position the GDEs as potent regulators of intercellular communication whose roles we are only beginning to understand.

The first connections between the GDEs and neurodevelopment came from experiments studying GDE2 in the embryonic chick (*Gallus gallus*). The developing chicken embryo is a longstanding model system of vertebrate neurodevelopment. Overexpression of GDE2 causes the precocious differentiation of progenitors into motor neurons (Rao and Sockanathan, 2005). GDE2 plays similar roles in the murine nervous system, indicating GDE2 has an evolutionarily conserved role between chick and mammalian neurodevelopment (Sabharwal et al., 2011; Rodriguez et al., 2012). Moreover, GDE2 has been implicated in oligodendrocyte maturation, neuroblastoma differentiation and motor neuron degeneration, indicating the broad necessity to regulate GPI-AP release in varied biological settings (Cave et al., 2017; Matas-Rico et al., 2017; Choi et al., 2020a; Nakamura et al., 2021). GDE3 has published roles in osteoblast differentiation and regulating the proliferation of oligodendrocyte precursor cells (OPCs) (Corda et al., 2009; Dobrowolski et al., 2020). The final member of the six-transmembrane GDEs is GDE6 (also called Gdpd4). GDE6 is implicated in spermatogenesis, however, its function(s) in the nervous system are undetermined (Nogusa et al., 2004; Fujihara et al., 2019). Using the caudal neural tube of the embryonic chick as our model system, we show that *Gde6* is strongly expressed by radial glia and its overexpression can influence crucial developmental processes including neuroepithelial expansion, dorsal-ventral (DV) patterning, and neurogenesis.

## Methods

### GDE6 plasmid amplification and purification

The pCAGGS:GDE6-FLAG construct was cloned using primers to amplify the coding region of chick *Gde6* (*Gdpd4*) from Stage 19 chick neural tube cDNA. The construct has an in frame 3x insertion of the Flag epitope (DYKDDDDK) before the stop codon. The pCAGGS vector uses the chicken β-actin promoter and has high expression in nervous system cell types. Successful insertion was confirmed with sanger-sequencing. pCAGGS:GDE6-FLAG and pCAGGS:empty-vector plasmid DNA was transformed into competent *E. coli* cells and purified using a nuclease free DNA purification kit (Qiagen).

### In-ovo electroporation

All animals were maintained and euthanized in accordance with the Middlebury College Institutional Animal Care and Use Committee. Fertilized, research grade chicken (*Gallus gallus*) eggs were obtained from Charles River Laboratories. The eggs acclimatized to room temperature for 2 h before incubation at 38°C and 50% relative humidity to the desired time point (Strombergs, INC1500). For electroporation, eggs were staged to Hamburger-Hamilton (HH) Stage 12 (∼48 h of incubation). To visualize the embryo, 5mL of egg white was withdrawn prior to windowing the egg, and a buffered ink solution (containing 135 mM NaCl, 2.6 mM KCl, 150 μM NaH_2_PO_4_⋅2H_2_O, 5.5 mM Glucose, 25 U/mL Penicillin-Streptomycin, 10% (v/v) India Ink) was injected into the yolk directly beneath the embryo. DNA solution (0.25–1 μg/μl of GDE6 plasmid DNA, 1% (w/v) Fast Green) was loaded into a fire pulled glass pipette. The needle was inserted into the caudal end of the neural tube and the ventricle was filled in the rostral direction until the dye reached the start of the rhombencephalon. The electroporation comprised five 50 ms pulses at 30V and was delivered by a Harvard Apparatus ECM 830 electroporator. Following electroporation, 1–3 drops of chilled antibiotic solution (25 U/mL Penicillin-Streptomycin) were added. Eggs were sealed with parafilm and incubated until the desired time point.

### Tissue preparation and immunohistochemistry

Embryos were dissected in cold phosphate-buffered saline (PBS) at the desired time point. Embryos were fixed in 4% paraformaldehyde in 0.1M Phosphate Buffer overnight at 4°C. Tissues were washed in PBS, cryoprotected in 30% sucrose overnight, and embedded in OCT Compound. Tissues were serially sectioned on a Leica CM 3050S cryostat at a thickness of 15 microns. Embedded and sectioned tissues were stored at –80°C. Tissue sections were stained using PBS + 0.03% Triton X-100 for permeabilization. Blocking was performed with 5% Normal Goat Serum. Primary antibodies: mouse anti-Flag (Sigma), rabbit anti-Olig2 (Millipore), rabbit anti-Sox2 (Millipore), mouse anti-Nkx2.2 (DSHB), mouse anti-FoxA2 (DSHB), mouse anti-Islet1/2 (DSHB), mouse anti-HB9 (DSHB). Corresponding secondary antibodies were used: goat anti-mouse Alexa Fluor and goat anti-rabbit Alexa Fluor (Jackson ImmunoResearch). Slides were coverslipped with mounting media containing DAPI (Vectashield).

### *In situ* hybridizations and RNAscope

An antisense oligonucleotide probe set targeting the 5′ Untranslated Region (UTR) of chicken (*Gallus gallus*) *Gde6* (also called *gdpd4*, NCBI Gene ID: 419084) was generated by ACDBio. Probe hybridization was performed according to the manufacturer’s protocol (ACDBio 323100-USM). Briefly, slides were washed with Phosphate Buffered Saline, post-fixed in 4% paraformaldehyde, and dehydrated in an ethanol series. Endogenous peroxidase activity was blocked with hydrogen peroxide solution, followed by high-temperature antigen retrieval. Tissue was treated with protease solution before incubation with RNAscope probes. Amplification ladders were sequentially annealed to the probe and Tyramide Sensitivity Assay (TSA) fluorophores (Perkin Elmer NEL744001KT) were used to visualize the probes. For RNAscope combined with immunohistochemistry, the primary antibody was applied overnight followed by a brief post-fixation with 10% Neutral Buffered Formalin prior to the protease treatment. The secondary antibodies were applied following the development of the TSA signal.

### Imaging and quantification

Images were taken using a Zeiss epifluorescent microscope or an Olympus laser-scanning FV3000 confocal microscope at 20× or 63×. Image acquisition settings were maintained between experimental conditions. Images were analyzed using ImageJ/FIJI (NIH). If an embryo had at least 3 sections where ≥50% of the dorsal-ventral extent of the neural tube had been successfully electroporated, it was included within the study for HH Stage 16 and 20. All forelimb level sections were included in the HH Stage 26 and control electroporation analyses. Figure legends indicate whether embryos were analyzed using a two-tailed *t*-test or a single factor ANOVA with repeated measures followed by *post-hoc* pairwise comparisons to assess significance. All statistical tests were run using Excel 2016 and SPSS with a significance level of *p* < 0.05. Error bars in the figures represent the standard error of the mean (SEM).

## Results

### *Gde6* expression during embryonic development

**FIGURE 1:**
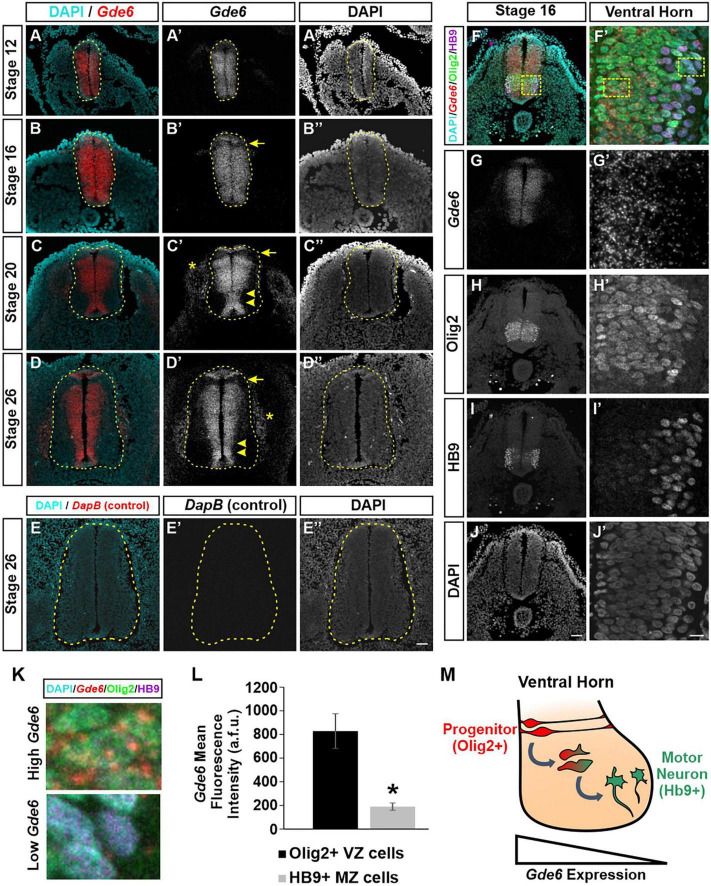
*Gde6* is expressed in the ventricular zone of the caudal neural tube. **(A–D”)** *In situ* hybridization using RNAscope shows robust *Gde6* expression in ventricular zone progenitors during patterning **(A,B)** and neurogenic **(C,D)** phases. Dashed yellow lines outline the neural tube, arrowheads highlight medial expression, arrows indicate a dorsal patterning domain that lacks *Gde6*, and asterisks denote expression in the periphery. **(E)** RNAscope negative control targeting bacterial *DapB* shows no signal. **(F–J’)** Combined RNAscope against *Gde6* and immunohistochemistry for progenitor marker Olig2 and motor neuron marker HB9. Insets in the right column are magnified from the dashed square in panel **(F)**. **(K)** Insets from panel **(F’)** illustrating high *Gde6-*expressing Olig2 + cells and low *Gde6*-expressing HB9 + cells. **(L)** Olig2 + cells have significantly higher *Gde6* expression compared to HB9 + motor neurons. Signal is quantified in arbitrary fluorescence units (a.f.u.). Unpaired two-tailed *t*-test, *p = 0.013, *n* = 4. Error bars represent S.E.M. **(M)** Summary diagram illustrating the decreasing expression of *Gde6* as cells transition from radial progenitors in the ventricular zone (VZ) to motor neurons in the marginal zone (MZ). Scale bar **(A–E”)** = 40 μm. Scale bar **(F–J)** = 40 μm. Scale bar **(F’–J’)** = 10 μm.

We first characterized the expression pattern of *Gde6* mRNA using RNAscope at several key stages during embryonic development. We chose time points in the caudal neural tube that reflect symmetrical radial glia proliferation and expansion (Stage 12), patterning and early neurogenesis (Stage 16 and 20), and late neurogenesis (Stage 26) (Hamburger and Hamilton, 1951). At stage 12, *Gde6* is expressed widely across the neural tube with reduced expression dorsally (Figures 1A–A”). By stage 16, expression is maintained across the broadening neurepithelium with the exception of the first DV patterning domain immediately ventral to the roof plate (Figures 1B–B”). As these radial glia proliferate, symmetric divisions expand the radial glia population, and asymmetric neurogenic divisions will produce one radial glia and one post-mitotic, immature neuron. Newly generated neurons will migrate laterally along the radial glia processes through the intermediate zone (IZ) to take up their terminal positions in the marginal zone (MZ). As more neurons are produced, the MZ expands to house these nascent neuronal populations creating the dorsal and ventral horns of the spinal cord. At stage 20, *Gde6* transcript becomes restricted to the VZ, indicating strong expression within the mitotic progenitor population (Figures 1C–C”, arrowheads). This medial expression pattern, including the floor plate and roof plate, becomes more pronounced by stage 26 as the MZ continues to expand across all of the DV domains (Figures 1D–D”). Notably, the absence of *Gde6* in the lone dorsal progenitor domain persists during through stage 26 (Figures 1C–D”, arrows). Further, *Gde6* is expressed in the periphery. Transcript is detected in mesodermal structures immediately adjacent to the neural tube; these are likely the dorsal root ganglia (Figures 1C–D”, asterisks). To evaluate the specificity of the *Gde6* probe, we performed the RNAscope hybridization using a probe targeting the bacterial gene *DapB* (Wang et al., 2012). As expected, no signal was detected from this negative control in the neural tube nor the periphery (Figures 1E–E”).

The exclusion of *Gde6* from post-mitotic regions in the lateral neural tube creates a gradient of expression from the VZ to the MZ. Based on the anatomical separation of cell types, we can infer that radial glia express *Gde6*, and that neuronal differentiation is associated with a downregulation of *Gde6*. To confirm this, we combined RNAscope, which allows single molecule detection, and immunohistochemistry for markers of medial pMN progenitors (Olig2) and lateral motor neurons (HB9) (Figures 1F–K; Rao and Sockanathan, 2005). The mean fluorescence intensity of *Gde6* within Olig2 + cells (828 ± 146 a.f.u.) was significantly higher than HB9 + cells (189 ± 31 a.f.u.) (Figure 1L). Taken together, our data indicate that *Gde6* is strongly expressed in radial glia progenitors in the caudal neural tube, and becomes starkly downregulated as daughter cells migrate to the MZ and differentiate into post-mitotic neurons (Figure 1M). Given the discrete expression of *Gde6* in radial glia, we hypothesized that GDE6 may regulate some of the functions of these progenitors, namely their ability to proliferate, specify within distinct DV patterning domains, and produce neurons. We will examine each of these processes in turn.

### GDE6 electroporation induces neural tube hyperplasia

**FIGURE 2:**
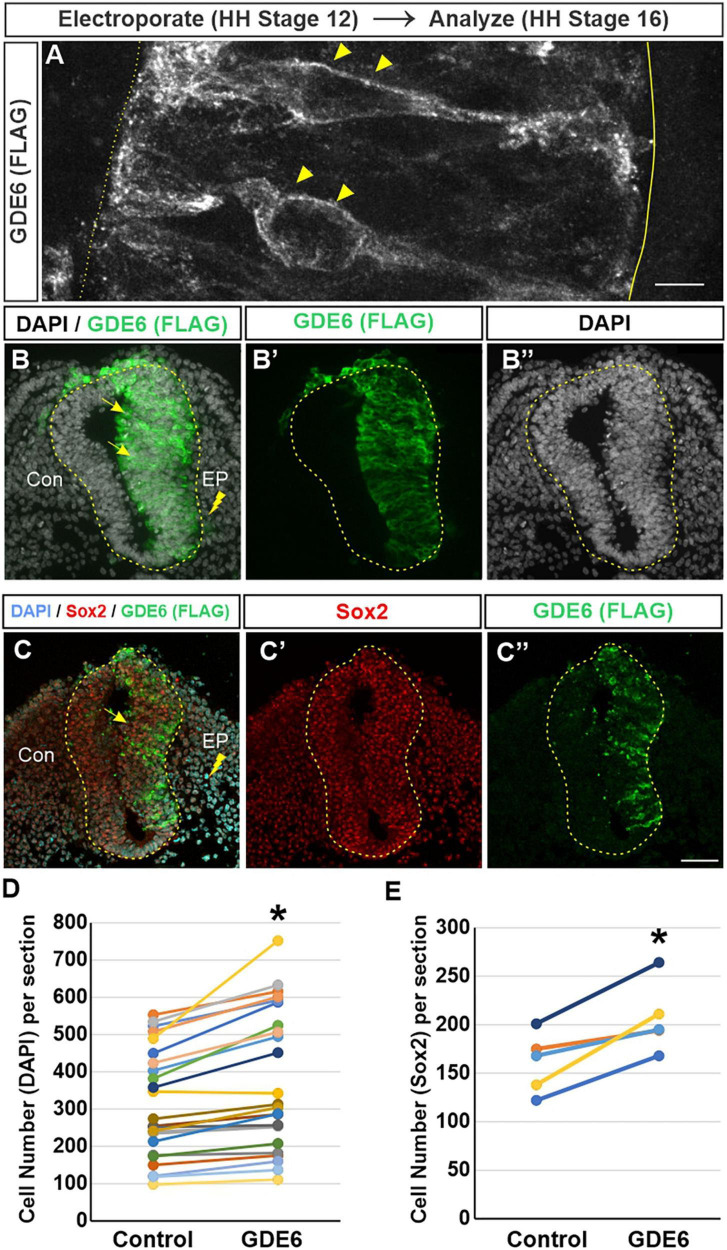
GDE6 overexpression induces neural tube hyperplasia. **(A–C”)** Transverse sections of stage 16 neural tube electroporated at Stage 12 with Flag-tagged GDE6. Anti-flag staining shows GDE6 expression in electroporated cells. **(A)** 63X confocal rendering of individual radial glia cells in the neurepithelium. GDE6 exhibits a membrane bound localization on the cell surface (arrowheads). Dotted line denotes the ventricular surface, solid line is the pial surface. **(B–C”)** Arrows denote the hyperplasia on the electroporated side (EP) compared to the control side (Con). DAPI labels all cell nuclei and Sox2 marks progenitors. **(B,C)** Are different embryos. **(D)** Quantification of neural tube cell number. Paired two-tailed *t*-test, *p < 0.001, *n* = 28. **(E)** Quantification of Sox2 + progenitors. Paired two-tailed *t*-test, *p = 0.011, *n* = 5. Scale bar **(A)** = 5 μm. Scale bar **(B–C’)** = 40 μm.

The advantage of the chick embryo is the ability to perform a unilateral in-ovo electroporation. This procedure introduces DNA constructs into the lumen of the neural tube followed by brief low-voltage electrical pulses that move the DNA into one half of the neural tube (Blank et al., 2007). Electroporated embryos will have an unchanged control side and a side that overexpresses GDE6. We expressed a C-terminal flag-tagged version of chicken GDE6 under control of the ubiquitously expressed β-actin promoter (pCAGGS:GDE6-FLAG). To confirm this construct’s ability to be expressed within radial glia, we electroporated stage 12 embryos and dissected them at stage 16. Using a low DNA concentration (0.25 μg/μl) we are able to achieve sparse labeling of electroporated cells. GDE6 protein can be visualized within individual radial glial cells on the electroporated half of the neural tube (Figure 2A). GDE6 protein is effectively trafficked to the plasma membrane, a necessary step to function as a cell-surface GPI-anchor cleaving enzyme. GDE6 is expressed somally and trafficked down both radial processes to the end-feet at the ventricular and pial surfaces (Figure 2A, arrowheads).

In fully electroporated embryos (1 μg/μl), GDE6 overexpression leads to a marked enlargement of the electroporated side compared to the control side (Figures 2B–B”). This expansion could result from one of, or a combination of, hypertrophy (an increase in cell size) or hyperplasia (an increase in cell number). We do not observe an overt increase in the area or density of individual DAPI + nuclei on the electroporated side, however, we find that the electroporated side has a 21% increase in the number of DAPI + cells compared to the control side (Figure 2D). To ensure that this increase reflects the expansion of progenitors as opposed to differentiated neurons, we stained for the transcription factor Sox2 (Figures 2C, E; Bylund et al., 2003). Additionally, electroporated neural tubes frequently contain masses of neuroepithelial cells ectopically located within the ventricle. These cells appear to be invaginations of the neurepithelium that have protruded into the ventricle, possibly as a consequence of the hyperplasia induced by GDE6 as these masses are Flag + (Supplementary Figure 1). Hyperplasia phenotypes were not observed in embryos electroporated with lower DNA concentration. Seen together, these data suggest that GDE6 overexpression positively regulates the number of progenitors in the caudal neural tube.

### GDE6 electroporation expands ventral progenitor patterning

**FIGURE 3:**
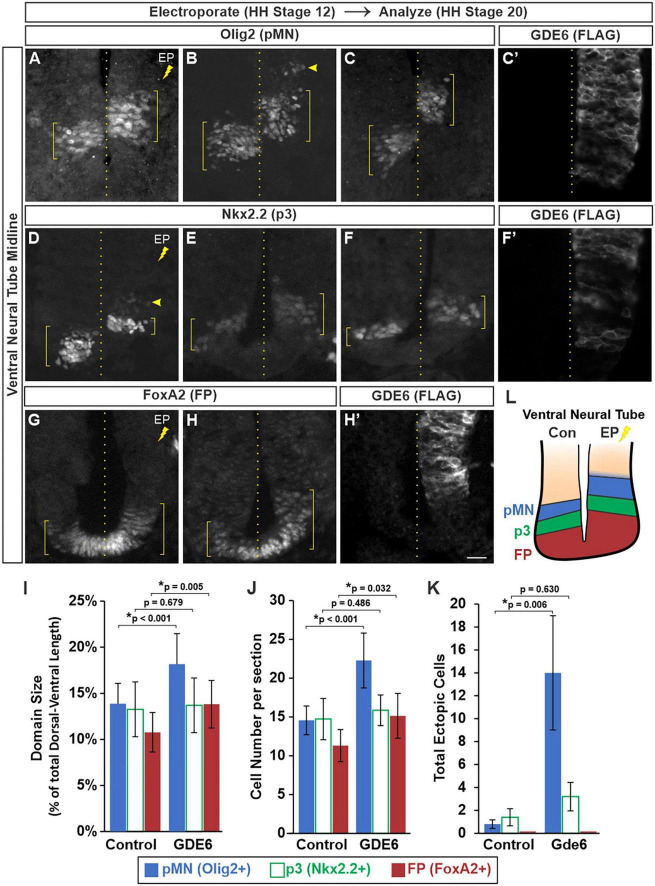
GDE6 regulates dorsal-ventral progenitor patterning. **(A–H’)** Transverse sections of stage 20 ventral neural tube electroporated at stage 12 with GDE6. Immunostaining for Olig2 **(A–C)**, Nkx2.2 **(D–F)**, and FoxA2 **(G,H)** visualize the pMN, p3 and floor plate, respectively. Each panel is a different embryo. **(C’,F’,H’)** Show adjacent sections stained for FLAG to visualize the extent of the electroporation for panels **(C,F,H)**. Brackets denote the dorsal-ventral extent of the progenitor domains, dotted line indicates the midline, and arrowheads highlight ectopic cells. **(I)** Quantification of the dorsal-ventral size of each patterning domain. One way ANOVA with repeated measures, *p < 0.001, *n* = 12. **(J)** Graph quantifying the mean number of cells per section in each domain. One way ANOVA with repeated measures, *p < 0.001, *n* = 12. **(K)** Quantification of the total number of ectopic cells detected outside of their respective domains. One way ANOVA with repeated measures, *p = 0.018, *n* = 8. No ectopic floor plate cells were detected. **(L)** Summary of the expanded ventral patterning domains following electroporation of GDE6. Error bars represent S.E.M. *Post-hoc* pairwise comparisons based on the estimated marginal means are displayed in the graphs. Scale bar **(A–H)** = 10 μm.

We next determined whether electroporated progenitors can effectively specify into their respective DV patterning domains. In stage 20 embryos that were electroporated at stage 12, we stained for transcription factors that label the floor plate (FoxA2), p3 (Nkx2.2), and pMN (Olig2) progenitor domains in the ventral neural tube. We detected a significant dorsal expansion of these progenitor domains on the electroporated side compared to control (Figures 3A–H’). To quantify these expansions we measured the dorsal-ventral extent of each domain (Figures 3A–H, brackets) as a percentage of the total DV length of the neural tube. This comparison will normalize variances caused by cryosectioning. The pMN comprised 13.8 ± 2.2% of the neural tube on the control side and 18.1 ± 3.3% on the electroporated side. The floor plate expanded from 10.7 ± 2.1% to 13.8 ± 2.5% (Figure 3I). The p3 domain did not have a significant change as its electroporated size was more variable, both contracting and expanding between biological replicates (compare 3D versus E). The changes in DV length were accompanied by increases in cell number with the pMN and FP showing a 53 and 33% increase, respectively, (Figure 3J).

Neighboring DV progenitor domains form sharp boundaries during development *via* genetic cross-repression by their transcription factors (Briscoe et al., 2000). This process ensures each progenitor is specified into a non-overlapping neuronal lineage. In contrast, the electroporated side contained ectopic Olig2 + cells situated dorsal to the pMN within the P2 domain (Figure 3B, arrowhead). The amount of ectopic Nkx2.2 + cells in the pMN was not significantly different than the control side, and no ectopic FoxA2 cells were detected in p3 (Figure 3K). These observations are consistent with an expansion of the FP and ventral progenitor identity upon overexpression of GDE6 (Figure 3L).

### Overexpression of GDE6 in radial glia reduces neurogenesis

**FIGURE 4:**
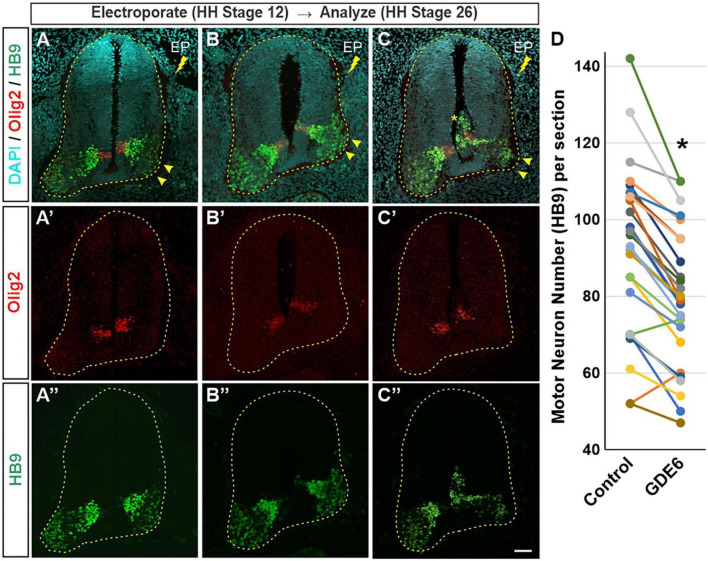
GDE6 overexpression disrupts neurogenesis. **(A–C”)** Transverse sections of stage 26 neural tube electroporated at stage 19 with Flag-tagged GDE6. Overexpression of GDE6 impairs the production motor neurons. The electroporated half of the neural tube has reduced numbers of HB9 + motor neurons and underdeveloped ventral horns (arrowheads). Asterisk indicates ectopic growth into the ventricular lumen. Dashed line outlines the neural tube. **(A–C)** Are taken from different embryos. **(D)** Quantification of HB9 + motor neuron numbers in the ventral horn comparing control versus GDE6 halves. Paired two-tailed *t*-test, *p < 0.001, *n* = 26. Scale bar **(A–C”)** = 40 μm.

Our data suggest that GDE6 works to maintain progenitor character by regulating neural tube size and dorsal-ventral patterning, and that down-regulation of GDE6 is correlated with neuronal differentiation. We therefore hypothesized that overexpression of GDE6 might decrease neurogenesis in the caudal neural tube. To test this notion, we electroporated GDE6 at stage 12 and waited until stage 26 to sacrifice the embryos. At this stage, neurogenesis is well underway with large populations of HB9 + motor neurons expanding the ventral horns. However, upon GDE6 overexpression, we see a significant reduction of post-mitotic neurons in the ventral horn on the electroporated side compared to control (Figures 4A–C”, arrowheads). Electroporated halves contain 16% fewer HB9 + cells compared to the control halves (Figure 4D). Moreover, ectopic ventricular masses contain HB9 + cells at this stage (Figure 4C, asterisk). These data reveal that the continued overexpression of GDE6 significantly reduces the production of post-mitotic neurons in the ventral horn.

Lastly, we performed a series of control experiments using the pCAGGS:empty-vector plasmid to ensure that our in-ovo electroporation technique alone does not produce any of the noted phenotypes. Crucially, we saw no differences in neural tube size, Olig2 patterning, or motor neuron differentiation, indicating that our phenotypes are specifically caused by GDE6 overexpression (Supplementary Figure 2).

## Discussion

Taken together, our experiments show that the GDE6 expression level can regulate three critical aspects of radial glia character: neural tube growth, cell specification, and the generation of post-mitotic daughter cells capable of neuronal differentiation. Chick embryos electroporated with GDE6 show an initial cellular hyperplasia and misdirected midline growth at stage 16 followed by imbalances in the proportions of the ventral DV patterning domains at stage 20. By stage 26, the ventral horn of the electroporated side is underdeveloped due to a reduced number of post-mitotic neurons. These observations coupled with its restricted expression pattern suggest that GDE6 and its downstream effectors play an important regulatory role in neural tube progenitors.

### GDE6 versus GDE2 and GDE3

The two other six-transmembrane GDE proteins (GDE2 and GDE3) have mutually exclusive expression patterns, with GDE2 expression in neurons and oligodendrocytes and GDE3 expression in oligodendrocyte precursor cells (OPCs) and astrocytes (Choi et al., 2020b; Dobrowolski et al., 2020). GDE6 is unique as it is the first of the six-transmembrane GDE proteins expressed in the nervous system. Interestingly, the *Gde6* expression pattern is complementary to *Gde2*, which begins as motor neurons differentiate in the MZ. They also appear to have a complementary function, as overexpression of GDE2 in the chick neural tube increases motor neuron number. With the expression of each GDE protein reinforcing a specific cell type, the transition from progenitor to neuron would necessitate the sharp transition from GDE6 to GDE2. These “hand-offs” are compelling regulatory checkpoints during neural tube development. Another occurs within the oligodendrocyte lineage as OPCs express GDE3 and mature oligodendrocytes switch to GDE2. GDE6, like GDE2 and GDE3, has been shown to be capable of GPI-anchor cleavage at the cell surface, however, they vary in substrate affinity (Park et al., 2013; Li et al., 2022). In this context, the cleavage state of cell surface GPI-anchors will change from a GDE6-dependent state to a GDE2-dependent state as differentiation proceeds.

GDE2 regulates differentiation non-cell autonomously *via* regulation of Notch signaling (Sabharwal et al., 2011; Park et al., 2013). The effect of GDE6 overexpression could work in a cell autonomous or non-cell autonomous manner, and our data raises the possibility of both actions. In favor of cell autonomous effects, the ectopic masses occurring on the electroporated side are invariably Flag + (Supplementary Figure 1A, asterisk). In addition, embryos expressing the lower DNA concentration that produces sparse labeling of radial glia do not display any hyperplasia. In favor of a non-cell autonomous effect, patterning shifts in the floor plate can occur even when those cells are not fully electroporated (Figures 3H–H”). Due to the transient nature of the overexpression, the Flag signal has largely dissipated by HH Stage 26 making cell autonomy conclusions difficult (Supplementary Figure 3). Moving forward, it will be crucial to identify the GPI-anchored substrates and signaling pathways affected by GDE6 overexpression.

Unlike GDE2, GDE3 and GDE6 are expressed in dividing cells. In OPCs, GDE3 negatively regulates the rate of proliferation by releasing Ciliary Neurotrophic Receptor Alpha (CNTFRα). OPC’s lacking GDE3 have a truncated S-phase (Dobrowolski et al., 2020). Strikingly, GDE6 appears to positively regulate the rate of proliferation in the early neurepithelium, at times prompting the ectopic expansion of hyperplasic tissue into the ventricle. In the future, a cell cycle analysis will be necessary to determine if GDE6 and GDE3 have congruent effects on proliferating cells. Other avenues that could lead to the GDE6-induced hyperplasia are a decrease in cell death and irregular rostral-caudal accumulation of radial glia. Given the narrow window between electroporation and dissection, the increased cell number is unlikely to be caused by a decrease in cell death. This developmental window is not typified by a high degree of apoptotic cell death, as is observed later in development during systems matching. Further, end-feet detachment leading to radial glia accumulations rostral-caudally is also unlikely, as this would yield the same net number of cells unevenly distributed among the tissue sections. Recent research has demonstrated that GDE2 expression levels can influence the growth of neuroblastoma cells. In this case, low levels of GDE2 expression are correlated with greater tumor growth and metastasis (Matas-Rico et al., 2016). In our system, high levels of GDE6 promote growths that are reminiscent of embryonal tumors. Ultimately, determining GDE6’s mode of action could be very relevant for other instances of unchecked growth and proliferation.

### The effect of GDE6 on patterning and neurogenesis

In neural tubes overexpressing GDE6 we observe a marked expansion of the FP and pMN accompanied by the ectopic localization of Olig2 + cells in the p2 domain. Considering *Gde6*’s mRNA expression in two key neural tube organizing centers (the floor plate and roof plate), it is possible that GDE6 affects morphogen release. Augmented Shh release from the floor plate and/or diminished TGF-β/Wnt signaling from the roof plate could produce the dorsal expansions (Lu et al., 2015). Alternatively, GDE6 might regulate morphogen transduction on the radial glia cell surface. For instance, GPI-anchored Gas1, a Shh co-receptor, could be a GDE6 substrate (Allen et al., 2011). In addition to the expansion of the pMN and FP, there is also an apparent shift of the domains toward the dorsal neural tube. The degree of this shift relative to the control side was not quantified as this difference can be influenced by sectioning, however, this shift is in line with the expanded size of the FP. The hyperplasia and patterning phenotypes may also produce a synergistic effect. The excess number of cells due to hyperplasia could act as a sink for released morphogens. Disrupting the DV morphogen gradients in this manner could produce disproportionate changes in the domain sizes. Future experiments should also address if the dorsal patterning domains display a commensurate contraction or loss of cross-repression.

GDE6 overexpression reduces the number of HB9 + cells and stunts ventral horn expansion, however, some motor neurons fail to migrate to the marginal zone and are ectopically located dorsal to the pMN or in ventricular masses (Figures 4B–C” and Supplementary Figure 1). Importantly, these migratory differences cannot fully explain the shrunken ventral horns, as many ventral horns are diminished without a nearby ventricular mass. Further, the quantification includes the ectopic masses on the electroporated side. Moving forward, it will be informative to characterize the migratory capacity of neurons produced from radial glia overexpressing GDE6.

### Expanded roles for GDE6

The six-transmembrane GDE proteins are emerging as an extremely versatile class of signaling proteins, likely due to the large diversity of GPI-anchored proteins and their broad expression inside and outside of the nervous system. Importantly, *Gde6* expression is conserved in higher vertebrates including mammals (Nogusa et al., 2004; The UniProt Consortium, 2014). Our findings provide an impetus for future loss-of-function studies and identify a unique and complementary role for GDE6 within its family of GPI-anchor cleaving enzymes.

## Data availability statement

The raw data supporting the conclusions of this article will be made available by the authors, without undue reservation.

## Ethics statement

The animal study was reviewed and approved by the Middlebury College Institutional Animal Care and Use Committee.

## Author contributions

MM performed the RNAscope experiments. FN and KB collected the hyperplasia dataset. TH, SS-C, and MK collected the patterning dataset. TJ and AW collected the neurogenesis data. FN, TJ, and MM performed the data analysis. CC designed the experiments, performed the data analysis, and wrote the manuscript. All authors contributed to the article and approved the submitted version.
