# Supplementary material for: GDE6 promotes progenitor identity in the vertebrate neural tube
Source: Front Neurosci. 2023 Mar 21;17:1047767. doi: 10.3389/fnins.2023.1047767 (PMC10070723; doi:10.3389/fnins.2023.1047767)
Supplement: Supplementary file 1 [file Presentation_1.pdf]

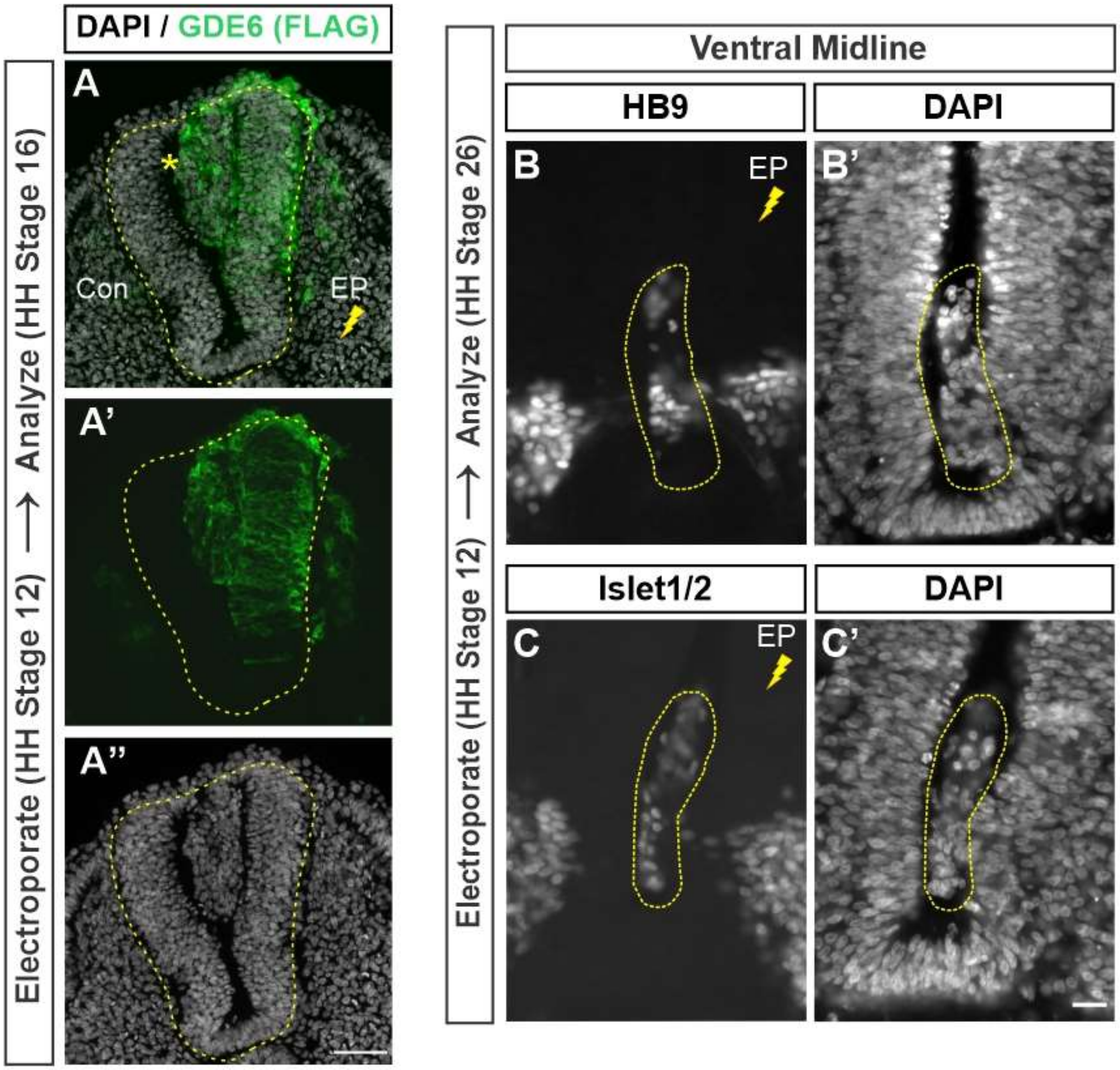

D Frequency of Embryos with Ectopic Neuroepithelial Cells	Stage 12 Electroporation		
	Stage 16	Stage 20	Stage 26
	25% (7/28 Embryos)	16% (2/12 Embryos)	15% (4/26 Embryos)

Figure S1: GDE6-electroporated embryos exhibit ectopic neuroepithelial protrusions
(A-C') Transverse sections of neural tubes electroporated at HH stage 12 and analyzed at HH stage 16 (A-A'') or HH Stage 26 (B-C'). Asterisk indicates an ectopic mass of neuroepithelial cells expanding into the lumen of the neural tube. **(B-C')** Ectopic midline growths (outlined) contain HB9+ and Islet1/2+ positive neurons. **(D)** Table summarizing the frequency of ectopic masses in electroporated embryos. Scale bar A-A'' = 40μm. Scale bar B-C' = 20μm.

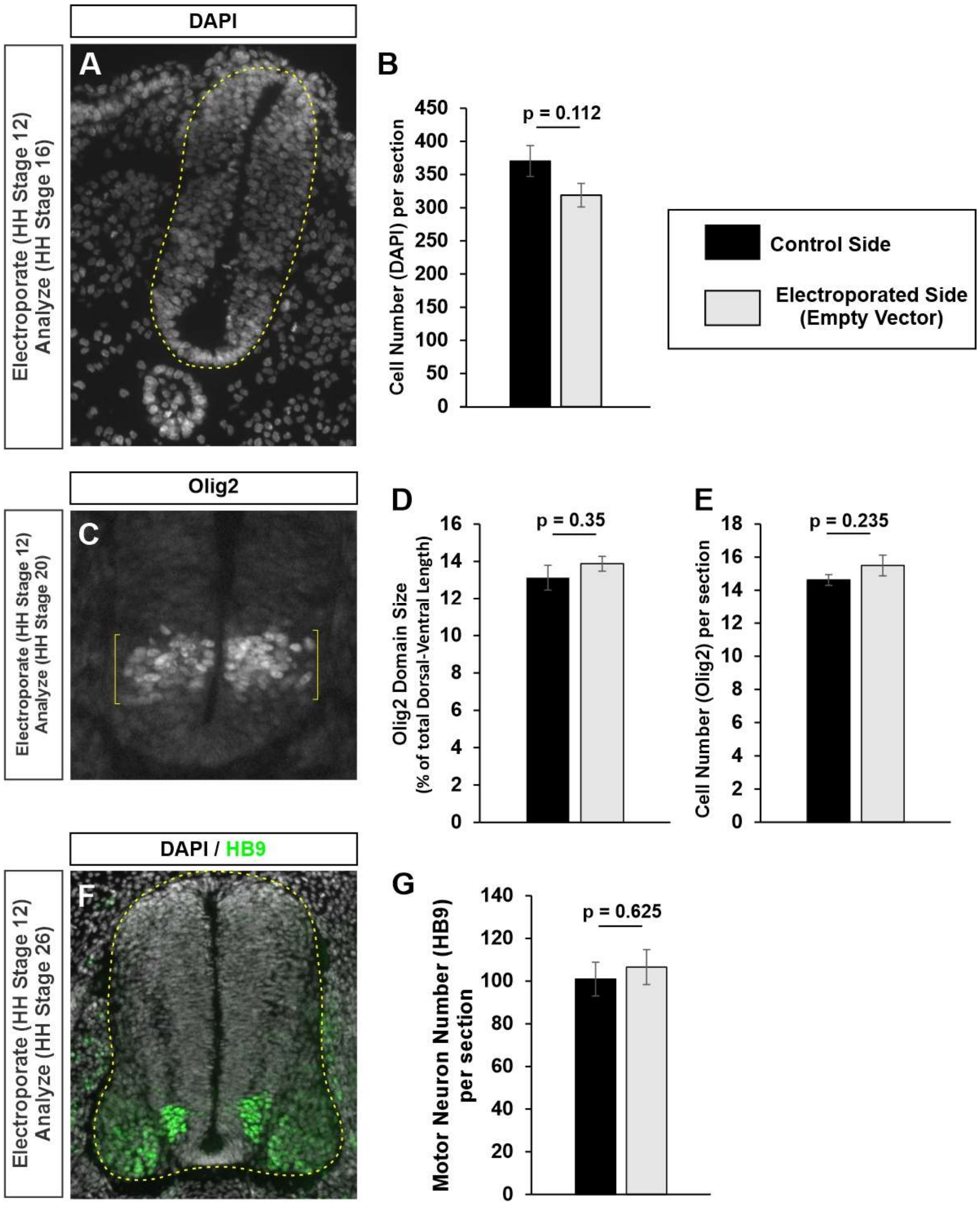

Figure S2. Control electroporations do not affect neuroepithelial size, Olig2 patterning, or motor neuron differentiation.

Control electroporations using the empty vector pCAGGS (1 μg/μl) plasmid produce no changes in neuroepithelial size (A,B), Olig2 patterning (C-E), or motor neuron differentiation (F,G). **(A, B)** At HH stage 16, control embryos do not exhibit any hyperplasia on the electroporated side. Unpaired two-tailed t test, p = 0.112, n = 10. **(C-E)** At HH stage 20, control embryos have no detectable asymmetries in the size of the pMN domain. Unpaired two-tailed t test, p = 0.35, n = 8. There is no significant difference in the number of Olig2+ cells in the pMN domain, Unpaired two-tailed t test, p = 0.235, n = 8. No ectopic cells were detected. Brackets denote the dorsal-ventral extent of the pMN domain. **(F, G)** At HH stage 26, control embryos show unaltered motor neuron differentiation. Each side has an equivalent number of HB9+ cells and clear ventrolateral expansion of the ventral horns. Unpaired two-tailed t test, p = 0.625, n = 11. Dashed line outlines the neural tube.

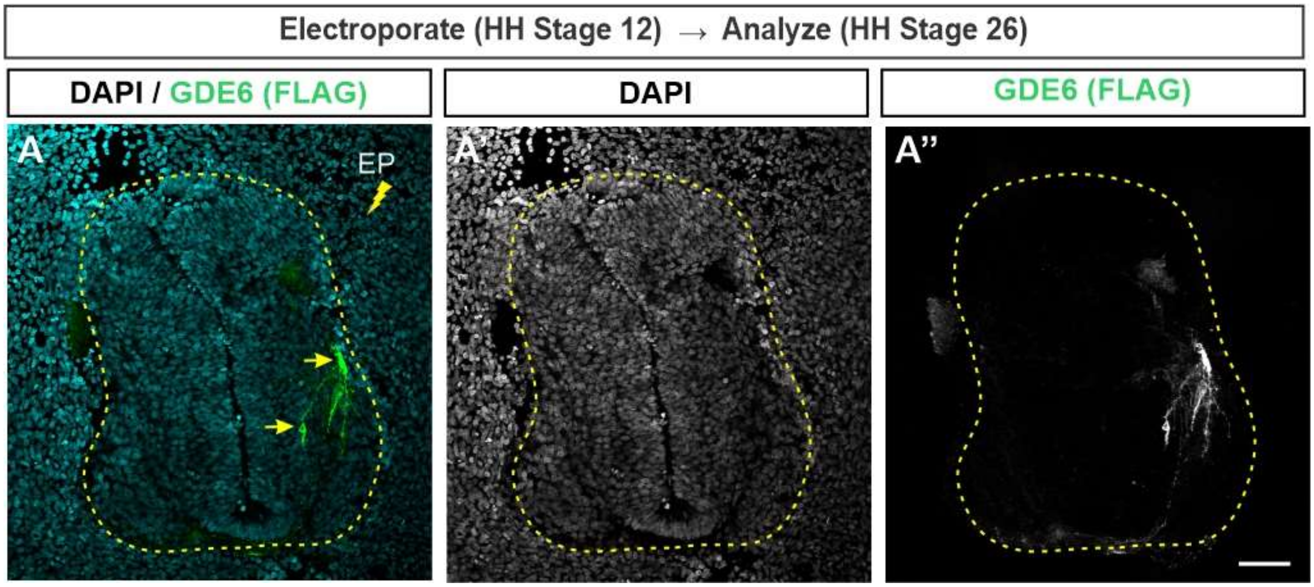

Figure S3. Minimal FLAG signal remains by HH Stage 26.

(A-A'') Transverse section of HH stage 26 embryos electroporated at HH Stage 12. As the GDE6 expression plasmid is not integrated into the genome, the degree of overexpression has largely waned by HH Stage 26. Arrows highlight labeled cells. This degree of FLAG staining prevents cell-autonomy conclusions at this stage; however, it still permits identification of the electroporated side. Dashed line outlines the neural tube. Scale bar = 40μm.
